# Cepharanthine Promotes Ca^2+^-Independent Premature Red Blood Cell Death Through Metabolic Insufficiency and p38 MAPK/CK1α/COX/MLKL/PKC/iNOS Signaling

**DOI:** 10.3390/ijms26157250

**Published:** 2025-07-27

**Authors:** Shaymah H. Alruwaili, Jawaher Alsughayyir, Mohammad A. Alfhili

**Affiliations:** Department of Clinical Laboratory Sciences, College of Applied Medical Sciences, King Saud University, Riyadh 12372, Saudi Arabia; 442204536@student.ksu.edu.sa (S.H.A.);

**Keywords:** cepharanthine, anticancer, eryptosis, hemolysis, p38 MAPK

## Abstract

Nonspecific toxicity to normal and malignant cells restricts the clinical utility of many anticancer drugs. In particular, anemia in cancer patients develops due to drug-induced toxicity to red blood cells (RBCs). The anticancer alkaloid, cepharanthine (CEP), elicits distinct forms of cell death including apoptosis and autophagy, but its cytotoxicity to RBCs has not been investigated. Colorimetric and fluorometric techniques were used to assess eryptosis and hemolysis in control and CEP-treated RBCs. Cells were labeled with Fluo4/AM and annexin-V-FITC to measure Ca^2+^ and phosphatidylserine (PS) exposure, respectively. Forward scatter (FSC) was detected to estimate cell size, and extracellular hemoglobin along with lactate dehydrogenase and aspartate transaminase activities were assayed to quantify hemolysis. Physiological manipulation of the extracellular milieu and various signaling inhibitors were tested to dissect the underlying mechanisms of CEP-induced RBC death. CEP increased PS exposure and hemolysis indices and decreased FSC in a concentration-dependent manner with prominent membrane blebbing. Although no Ca^2+^ elevation was detected, chelation of intracellular Ca^2+^ by BAPTA-AM reduced hemolysis. Whereas SB203580, D4476, acetylsalicylic acid, necrosulfonamide, and melatonin inhibited both PS exposure and hemolysis, staurosporin, L-NAME, ascorbate, caffeine, adenine, and guanosine only prevented hemolysis. Interestingly, sucrose had a unique dual effect by exacerbating PS exposure and reversing hemolysis. Of note, blocking KCl efflux augmented PS exposure while aggravating hemolysis only under Ca^2+^-depleted conditions. CEP activates Ca^2+^-independent pathways to promote eryptosis and hemolysis. The complex cytotoxic profile of CEP can be mitigated by targeting the identified modulatory pathways to potentiate its anticancer efficacy.

## 1. Introduction

Anemia is one of the most common complications in cancer patients, and overwhelming evidence implicates eryptosis and hemolysis in chemotherapy-associated anemia [1,2]. Eryptosis is a physiological process that eliminates damaged erythrocytes without rupturing the cell membrane and releasing inflammatory contents. Cellular changes during eryptosis include loss of volume, appearance of blebs in the cell membrane, and phosphatidylserine (PS) exposure. These PS molecules are identified and bound to phagocytes to rid the bloodstream of dysfunctional cells. Eryptosis is under regulation by a growing repertoire of signaling mediators, most importantly, casein kinase 1α (CK1α), p38 mitogen-activated protein kinase (p38 MAPK), and protein kinase C (PKC). Calcium mobilization constitutes the main mechanism of cell death in RBCs, in addition to acetylcholinesterase (AChE) inhibition, ceramide nucleation in the cell membrane, oxidative stress, and energy deficit [3].

**Figure 1:**
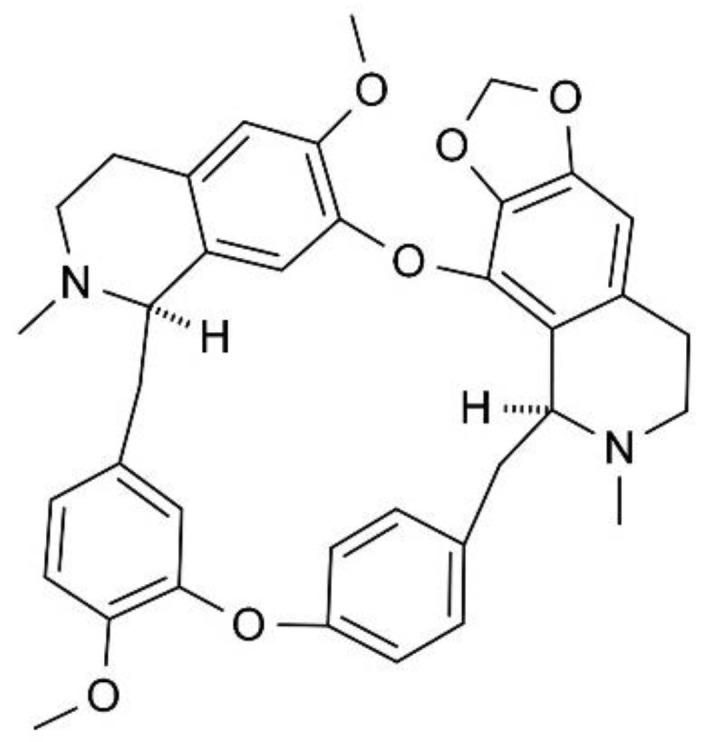
Molecular structure of CEP. The bisbenzylisoquinoline units are noted.

Cepharanthine (CEP) is a biscoclaurine alkaloid (Figure 1) in the tuberous geophyte *Stephania cephalantha*. In addition to its anti-inflammatory, antioxidant, and antimicrobial properties [4], accumulating evidence suggests that CEP has anticancer activity demonstrated in renal [5], breast [6], gastric [7], ovarian [8], colorectal [9], nasopharyngeal [10], liver [11], and lung [12] cells. CEP particularly targets apoptosis and autophagy as cell death modalities to exert its anticancer effects, and multiple mechanisms have been described. These include cell cycle arrest, oxidative stress, transcriptional perturbation through miRNA, caspase stimulation, and inhibition of translation, migration, invasion, and angiogenesis [4].

Efforts continue to uncover the range of eryptosis modulators among candidate anticancer compounds [13], but further research is needed to assess the hematological toxicity of CEP. The objective of the current study is to investigate the eryptotic and hemolytic properties and biochemical mechanisms of CEP in human erythrocytes.

## 2. Results

### 2.1. CEP Promotes Eryptosis

**Figure 2:**
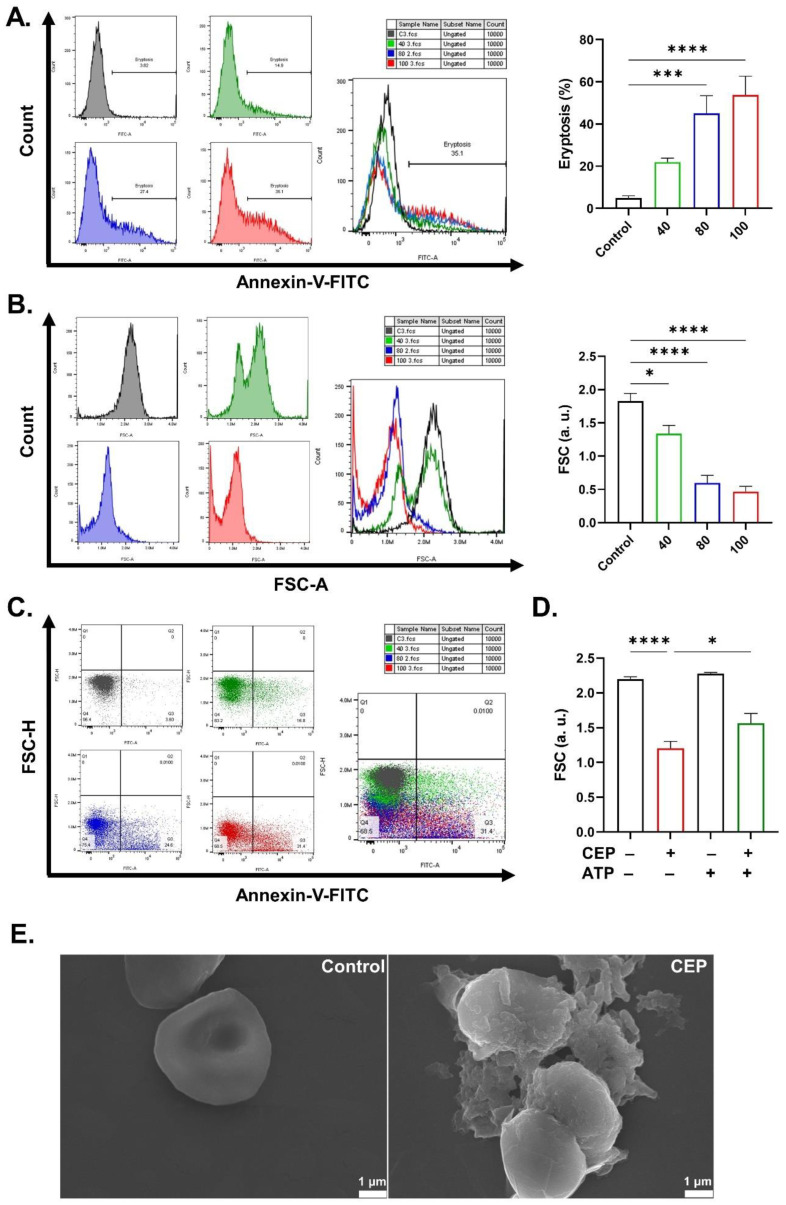
Stimulation of eryptosis by CEP. (**A**) Original histograms of eryptotic cells. (**B**) Original histograms of cell volume. (**C**) Original dot plots of FSC-H vs. annexin-V-FITC. (**D**) FSC in arbitrary units in control and CEP-treated (100 μM) cells with and without ATP (0.5 mM). (**E**) SEM analysis (×7000) showing loss of the biconcave shape with a coarse surface and prominent membrane blebs in CEP-treated cells. Results are shown as means ± SEM (*n* = 9) as analyzed by one-way ANOVA. * (*p* < 0.05), *** (*p* < 0.001), and **** (*p* < 0.0001). CEP concentrations: 40, 80, and 100 μM. Incubation time: 48 h.

The objective of the current study is to assess the cytotoxicity of CEP in RBCs. To examine eryptosis, we measured PS exposure and FSC, and we found that CEP significantly increased the amount of annexin-positive cells (Figure 2A) at 80 (*p* < 0.001) and 100 μM (*p* < 0.0001) and decreased FSC (Figure 2B) at 40 (*p* < 0.05), 80 (*p* < 0.0001), and 100 μM (*p* < 0.0001). The distribution of cells based on their viability and volume is depicted in Figure 2C. Interestingly, supplementation of the culture medium with ATP attenuated CEP-induced cellular dehydration (*p* < 0.05; Figure 2D). To confirm eryptosis, SEM inspection revealed a marked loss of the biconcave shape with spheroid cells exhibiting a coarse, uneven surface with prominent membrane blebs (Figure 2E).

### 2.2. CEP Promotes Hemolysis

**Figure 3:**
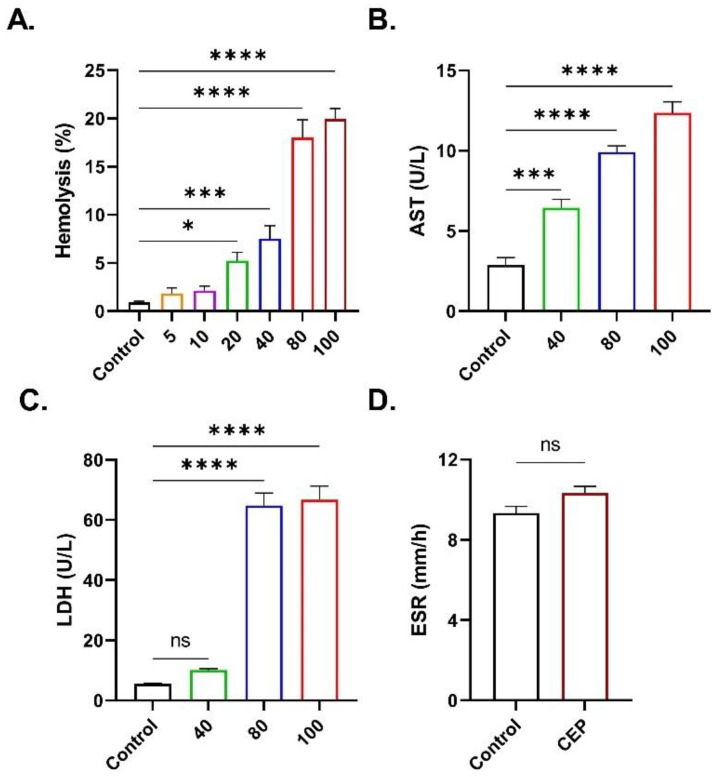
Stimulation of hemolysis by CEP. (**A**) Hemolysis. (**B**) LDH and (**C**) AST activities in supernatants. (**D**) ESR (100 μM). Results are shown as means ± SEM (*n* = 9) as analyzed by one-way ANOVA (*t*-test for ESR). * (*p* < 0.05), *** (*p* < 0.001), and **** (*p* < 0.0001). CEP concentrations: 5–100 μM. Incubation time: 48 h.

In Figure 3A, it is shown that CEP also induced significant hemolysis at 20 (*p* < 0.05), 40 (*p* < 0.001), 80 (*p* < 0.0001), and 100 μM (*p* < 0.0001) with concomitant leakage of AST (Figure 3B) and LDH (Figure 3C). Nevertheless, no significant increase in ESR was detected (Figure 3D).

### 2.3. CEP Cytotoxicity Is Independent of Ca^2+^ or Oxidative Stress

**Figure 4:**
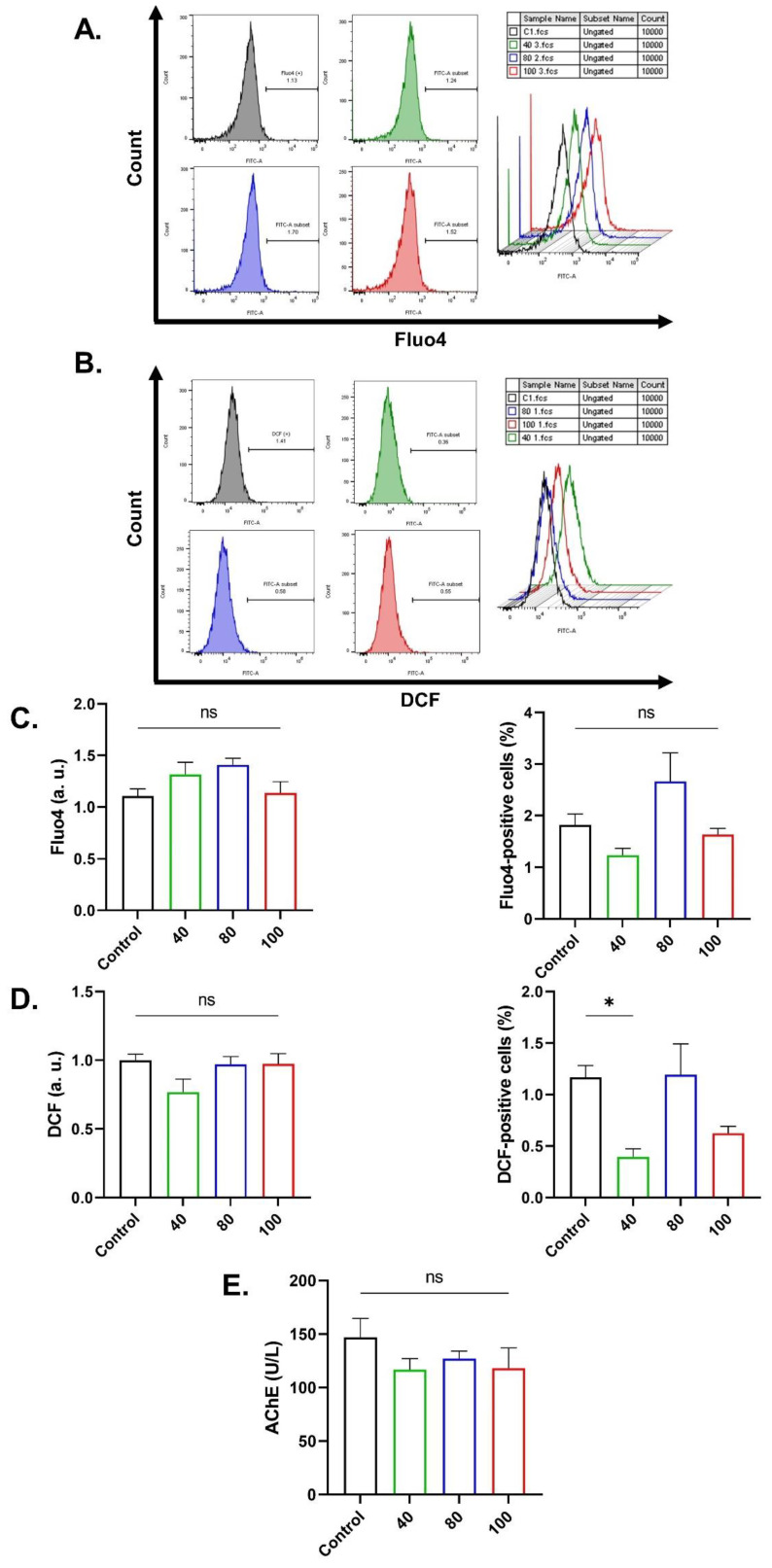
CEP cytotoxicity is independent of Ca^2+^ mobilization and redox imbalance. (**A**) Original histograms of Fluo4 fluorescence. (**B**) Original histograms of DCF fluorescence. (**C**) Fluo4 fluorescence and percentage of Fluo4-positive cells. (**D**) DCF fluorescence and percentage of DCF-positive cells. (**E**) AChE activity in hemolysates. Results are shown as means ± SEM (*n* = 9) as analyzed by one-way ANOVA. No statistical significance is indicated by ns while * (*p* < 0.05) is shown. CEP concentrations: 40, 80, and 100 μM. Incubation time: 48 h.

**Figure 5:**
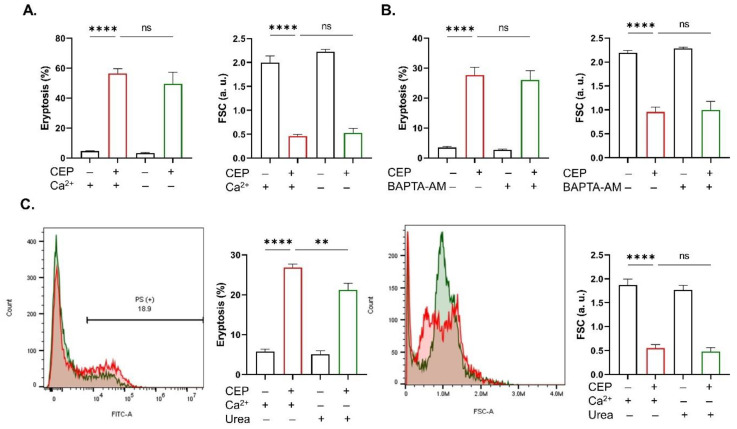
Eryptosis inhibitors. Eryptosis and FSC with and without (**A**) extracellular Ca^2+^, (**B**) BAPTA-AM (10 μM), and (**C**) extracellular Ca^2+^ and urea (25 mM). Results are shown as means ± SEM (*n* = 9) as analyzed by one-way ANOVA. No statistical significance is indicated by ns while ** (*p* < 0.01) and **** (*p* < 0.0001) are shown. CEP concentration: 100 μM. Incubation time: 48 h.

Although Ca^2+^ mobilization and redox imbalance are integral mechanisms underlying eryptosis and hemolysis, CEP toxicity (40, 80, and 100 μM) was not associated with neither Ca^2+^ elevation nor oxidative stress, and there was also no significant inhibition of AChE upon CEP exposure, as revealed in Figure 4. Congruently, exclusion of extracellular Ca^2+^ (Figure 5A) and chelation of intracellular Ca^2+^ by BAPTA-AM (Figure 5B) had no appreciable effect on neither PS exposure nor FSC. However, removal of Ca^2+^ and addition of urea together significantly inhibited PS exposure (*p* < 0.01) without affecting FSC (Figure 5C).

### 2.4. Inhibitor of CEP-Induced Hemolysis

**Figure 6:**
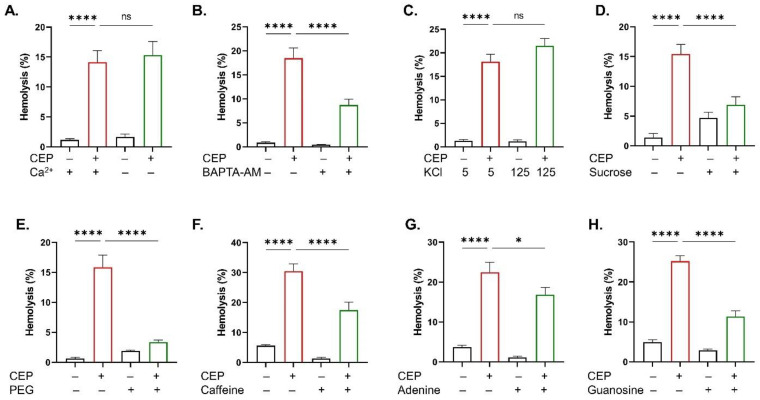
Hemolysis inhibitors. Hemolysis with and without (**A**) extracellular Ca^2+^, (**B**) BAPTA-AM (10 μM), (**C**) 5 and 125 mM KCl, (**D**) sucrose (40 mM), (**E**) PEG (10% *v*/*v*), (**F**) caffeine (0.5 mM), (**G**) adenine (2 mM), and (**H**) guanosine (2 mM). Results are shown as means ± SEM (*n* = 9) as analyzed by one-way ANOVA. No statistical significance is indicated by ns while * (*p* < 0.05) and **** (*p* < 0.0001) are shown. CEP concentration: 100 μM. Incubation time: 48 h.

In the case of hemolysis, Ca^2+^ entry was not important to CEP action (Figure 6A), but cytoplasmic Ca^2+^ chelation significantly blunted CEP-induced hemolysis (*p* < 0.0001; Figure 6B). Likewise, although K^+^ efflux did not seem to be essential to the hemolytic activity of CEP (Figure 6C), co-treatment with sucrose (*p* < 0.0001; Figure 6D), PEG (*p* < 0.0001; Figure 6E), caffeine (*p* < 0.0001; Figure 6F), adenine (*p* < 0.05; Figure 6G), and guanosine (*p* < 0.0001; Figure 6H) significantly inhibited hemolysis.

### 2.5. KCl Enrichment and Sucrose Exacerbate CEP-Induced Eryptosis

**Figure 7:**
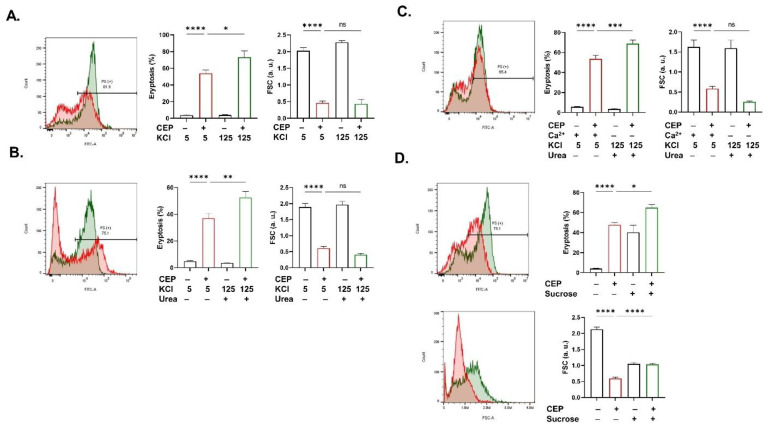
Eryptosis-sensitizing conditions. Eryptosis and FSC with and without (**A**) 5 and 125 mM KCl, (**B**) 5 and 125 mM KCl in presence of urea (25 mM), (**C**) 5 and 125 mM KCl in presence of urea (25 mM) and absence of Ca^2+^, and (**D**) sucrose (40 mM). Results are shown as means ± SEM (*n* = 9) as analyzed by one-way ANOVA. No statistical significance is indicated by ns while * (*p* < 0.05), ** (*p* < 0.01), *** (*p* < 0.001), and **** (*p* < 0.0001) are shown. CEP concentration: 100 μM. Incubation time: 48 h.

Notably, some shared and distinct physiological manipulations were found to exacerbate CEP-induced eryptosis and hemolysis. PS exposure was significantly increased under high KCl conditions while FSC was not affected (*p* < 0.05; Figure 7A). The same outcome was observed in the presence of urea (*p* < 0.01; Figure 7B) and in the absence of Ca^2+^ from the extracellular space (*p* < 0.001; Figure 7C), both while preventing K^+^ loss. In contrast, sucrose aggravated both PS exposure (*p* < 0.05) and cell shrinkage (*p* < 0.0001) as seen in Figure 7D.

### 2.6. Urea and KCl Enrichment Sensitize RBCs to the Hemolytic Activity of CEP

**Figure 8:**
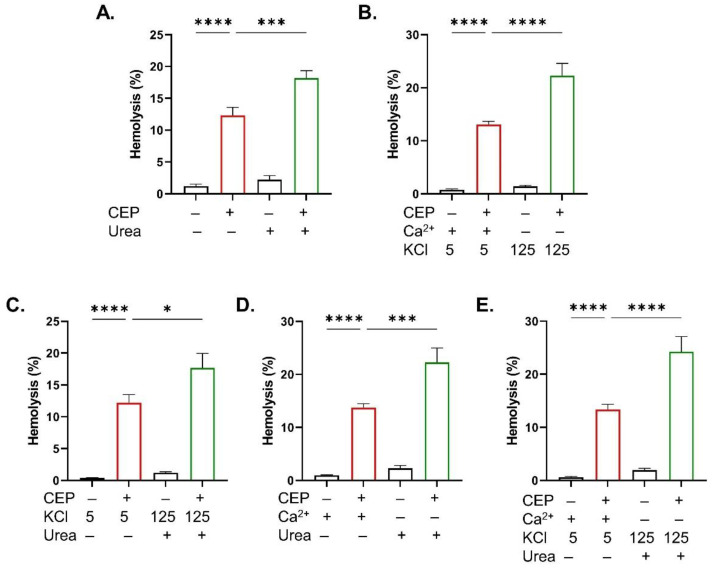
Hemolysis-sensitizing conditions. Hemolysis with and without (**A**) urea (25 mM), (**B**) 5 and 125 mM KCl in absence of Ca^2+^, (**C**) urea (25 mM) in absence of Ca^2+^, (**D**) 5 and 125 mM KCl in presence of urea (25 mM), and (**E**) 5 and 125 mM KCl in presence of urea (25 mM) and absence of Ca^2+^. Results are shown as means ± SEM (*n* = 9) as analyzed by one-way ANOVA. * (*p* < 0.05), *** (*p* < 0.001), and **** (*p* < 0.0001). CEP concentration: 100 μM. Incubation time: 48 h.

Hemolysis, on the other hand, was significantly aggravated by urea (*p* < 0.001; Figure 8A) and by Ca^2+^-depleted conditions supplemented with either high KCl (*p* < 0.0001; Figure 8B) or urea (*p* < 0.05; Figure 8C). Furthermore, the addition of urea to the K^+^-rich medium either in the presence (*p* < 0.001; Figure 8D) or absence (*p* < 0.0001; Figure 8E) of Ca^2+^ significantly potentiated CEP-induced hemolysis.

### 2.7. Signaling Cascades Involved in CEP Cytotoxicity

**Figure 9:**
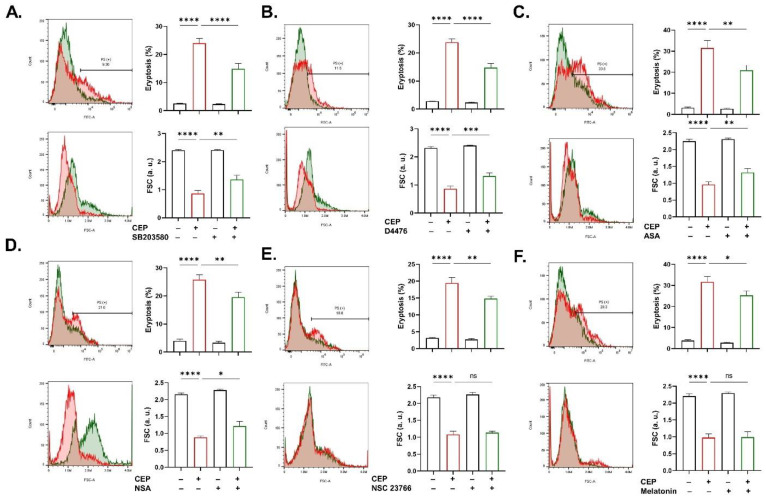
Intracellular signaling cascades governing CEP-induced eryptosis. Eryptosis and FSC with and without (**A**) SB203580 (100 μM), (**B**) D4476 (20 μM), (**C**) ASA (25 μM), (**D**) NSA (0.5 μM), (**E**) NSC 23766 (100 μM), and (**F**) melatonin (1 μM). Results are shown as means ± SEM (*n* = 9) as analyzed by one-way ANOVA. No statistical significance is indicated by ns whereas * (*p* < 0.05), ** (*p* < 0.01) *** (*p* < 0.001), and **** (*p* < 0.0001) are shown. CEP concentration: 100 μM. Incubation time: 48 h.

**Figure 10:**
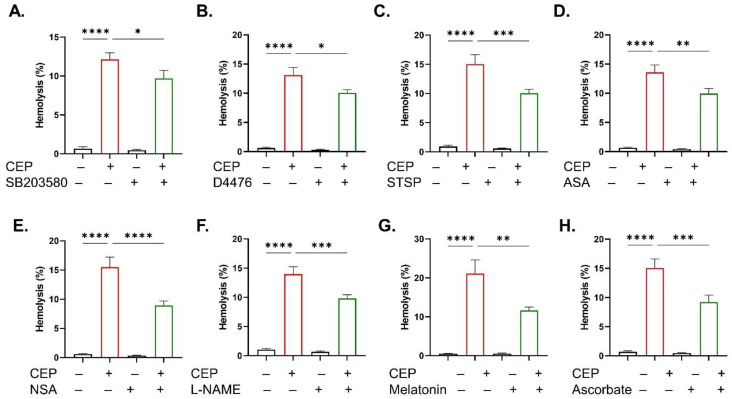
Signal transduction analysis of CEP-induced hemolysis. Hemolysis with and without (**A**) SB203580 (100 μM), (**B**) D4476 (20 μM), (**C**) STSP (1 μM), (**D**) ASA (25 μM), (**E**) NSA (0.5 μM), (**F**) L-NAME (20 μM), (**G**) melatonin (1 μM), and (**H**) ascorbate (1 mM). Results are shown as means ± SEM (*n* = 9) as analyzed by one-way ANOVA. * (*p* < 0.05), ** (*p* < 0.01) *** (*p* < 0.001), and **** (*p* < 0.0001). CEP concentration: 100 μM. Incubation time: 48 h.

Signal cascade evaluation identified several mediators driving CEP-induced eryptosis. PS exposure and FSC were both attenuated in the presence of SB203580 (*p* < 0.0001 and *p* < 0.01; Figure 9A), D4476 (*p* < 0.0001 and *p* < 0.001; Figure 9B), ASA (*p* < 0.01 and *p* < 0.01; Figure 9C), and NSA (*p* < 0.01 and *p* < 0.05; Figure 9D), whereas only PS exposure was ameliorated by NSC 23766 (*p* < 0.01; Figure 9E) and melatonin (*p* < 0.05; Figure 9F). Likewise, the hemolytic potential of CEP was significantly suppressed by SB203580 (*p* < 0.05; Figure 10A), D4476 (*p* < 0.05; Figure 10B), STSP (*p* < 0.001; Figure 10C), ASA (*p* < 0.01; Figure 10D), NSA (*p* < 0.0001; Figure 10E), L-NAME (*p* < 0.001; Figure 10F), melatonin (*p* < 0.01; Figure 10G), and ascorbate (*p* < 0.001; Figure 10H). Of note, several physiological manipulations and inhibitors did not show significant effects on neither eryptosis (Figure S1) nor hemolysis (Figure S2).

## 3. Discussion

The lack of tissue specificity of prospective anticancer compounds severely undermines their therapeutic applications. This study offers experimental evidence of the cytotoxicity of CEP in RBCs and highlights the complexity of the underlying mechanisms and signaling networks involved (Table 1). Because chemotherapy-related anemia can be caused by both eryptosis and hemolysis [1,2], the revealed pathways constitute an important addition to current understanding of CEP as an anticancer compound. In particular, pharmacological targeting of these pathways using specific inhibitors may enhance the therapeutic specificity of CEP to tumor cells by mitigating hematologic side effects. Lead candidates, including SB203580, D4476, ASA, NSA, STSP, and L-NAME, warrant further investigation for their ability to selectively block eryptosis without affecting anticancer pathways. The potential application of inhibitors as adjuvants in combination therapy relies on dissecting these overlapping or synergistic interactions to develop safer, more targeted CEP-based regimens.

The collapse of membrane asymmetry follows flippase inactivation and scramblase activation which switches PS molecules from inside to outside the cell (Figure 2). Surveillance phagocytes express PS receptors and bridging molecules in order to adhere to and engulf eryptotic cells. Eventually, the number of circulating RBCs becomes insufficient to sustain oxygen delivery as cells undergoing eryptosis are prematurely eliminated. This is particularly relevant to cancer in addition to a wide array of chronic diseases and infections [3]. Of particular interest is malaria, in which *Plasmodium* parasites trigger eryptosis through a variety of mechanisms including Ca^2+^ entry, oxidative stress, and ceramide accumulation. This host defense mechanism aims to limit parasitemia by enhancing the clearance of infected cells [14]. Therefore, eryptosis inducers, including CEP, may help augment this response. Indeed, further studies are needed to explore the potential of CEP as a complementary agent in malaria treatment.

Beside anemia, other important consequences of eryptosis include hypercoagulability, thrombosis, and exacerbation of inflammation [15]. Interaction with eryptotic cells also depletes nitric oxide (NO) which impairs vasodilation and endothelial function [16]. Notably, co-treatment of cells with L-NAME significantly reduces hemolysis (Figure 10) indicating the involvement of NOS in CEP toxicity. This enzyme replenishes NO and CEP which may therefore activate NO signaling to induce membrane rupture. Nitrosative stress and formation of NO-derived reactive species such as peroxynitrite may be one of the mechanisms mediating the hemolytic activity of CEP.

Interestingly, Ca^2+^ mobilization, the most important mechanism of eryptosis, is apparently not targeted by CEP (Figure 4). Accordingly, Ca^2+^ elimination did not ameliorate neither eryptosis (Figure 5) nor hemolysis (Figure 6). However, the presence of urea under Ca^2+^-depleted conditions prevented eryptosis (Figure 5) while urea alone had no appreciable effect (Figure S1). This suggests that, when Ca^2+^ influx is eliminated, scramblase remains sufficiently inactive, and urea simultaneously modulates membrane fluidity, cytoskeleton arrangement, or protein conformation, which then stabilizes the bilayer asymmetry [17]. An opposite effect on hemolysis was, however, observed, again irrespective of Ca^2+^ availability or KCl efflux (Figure 8), indicating osmotic swelling due to water accumulation.

Several anti-hemolytic compounds were identified including BAPTA-AM, sucrose, and PEG (Figure 6). The fact that BAPTA-AM, as opposed to extracellular Ca^2+^ depletion, was effective strongly suggests that intracellular Ca^2+^ signaling, but not Ca^2+^ influx, acts upstream of membrane–cytoskeleton disruption even if levels do not rise measurably (Figure 4). The contrasting effects of sucrose on hemolysis and PS exposure (Figure 7) are due to the non-penetrating nature of this sugar. Sucrose induces remarkable shrinkage as it generates an osmotic gradient between the medium and the intracellular environment which is a strong trigger of eryptosis. The prevention of hemolysis indicates that CEP may recruit water inflow and cell swelling which sucrose counteracts due to its osmotic activity. Mechanical damage as a potential mechanism of CEP-induced hemolysis is also proposed by the protective effect of PEG. This compound can insert into the membrane and seal lesions and pores [18], possibly formed by MLKL [19] (Figure 10). Thus, implications of CEP PEGylation for drug delivery and overall efficacy deserve further investigation.

Caffeine has been shown to prevent eryptosis [20] and hemolysis [21] and to both promote and inhibit apoptosis [22,23]. In the presence of CEP, caffeine reduces hemolysis (Figure 6) probably through phosphodiesterase inhibition, an anti-inflammatory role, or modulation of glucose metabolism [24]. This is further supported by the fact that COX inhibition by ASA (Figure 10), along with metabolic replenishment by energy substrates, adenine and guanosine (Figure 6), similarly ameliorate CEP-induced hemolysis. Importantly, ATP was only effective against cell dehydration (Figure 2), suggesting that CEP perturbs mediators upstream of ATP synthesis to trigger PS exposure and membrane rupture. Further analysis of the specific targets within the glycolytic pathway is likely to reveal the identity of these mediators.

The most effective inhibitors of CEP toxicity were SB203580, D4476, ASA, and NSA, as they significantly attenuated PS exposure, cell shrinkage, and hemolysis (Figure 9 and Figure 10). Altogether, this observation identifies a signaling axis consisting of p38 MAPK, CK1α, COX, and MLKL as the major underlying mechanism accounting for the cytotoxicity of CEP in RBCs. In erythrocytes, stress response is orchestrated by p38 MAPK [25], survival by CK1α [26] and COX [27], and necroptosis by MLKL [28]. Numerous complex interactions among these mediators have previously been described in the literature. Moreover, Ca^2+^-independent chloride efflux stimulates COX, which is in parallel under regulation by MAPK, to induce eryptosis. Similarly, MLKL functions as the executioner protein of necroptosis, but detection of the necroptosome assembly is needed to confirm the stimulation of this death pathway.

Rac1 GTPase is uniquely attributed to the eryptotic (Figure 9) but not hemolytic (Figure S2) activity of CEP as shown by NSC 23766. This observation points at the participation of Rac1 signaling in phospholipid rearrangement possibly through modulation of the cytoskeleton [29] which has been shown to facilitate *Plasmodium* invasion [30]. Targeting Rac1, therefore, offers a novel therapeutic strategy against a multitude of pathological conditions involving erythrocytes.

Despite no evidence of redox imbalance (Figure 4), our findings indicate that PS exposure and hemolysis are significantly blunted by melatonin while hemolysis was significantly inhibited by ascorbic acid (Figure 9 and Figure 10). While both melatonin and ascorbic acid function as powerful antioxidants [31,32], their observed protection against CEP toxicity is unlikely to be attributed to their role as free radical scavengers. Instead, both may inhibit stress-related signaling pathways independently of free radicals, which is supported by their reported non-canonical functions [33,34], although transient elevations in free radicals at an early time point cannot be ruled out.

Several modifications to the incubation medium paradoxically potentiated CEP toxicity. In the case of eryptosis, the major mechanism that exacerbated PS exposure was the blockage of K^+^ efflux by increasing extracellular KCl from 5 to 125 mM, regardless of Ca^2+^ absence or urea availability (Figure 7), which were both required in tandem to prevent PS exposure (Figure 5). Such a finding indicates that, when CEP is unable to stimulate K^+^ efflux, other mechanisms are activated which leads to augmented cellular damage. These mechanisms are likely to involve one or more of the identified mediators and pathways in this work. In contrast, while neither elimination of extracellular Ca^2+^ or blockade of K^+^ loss alone was sufficient to mitigate hemolysis (Figure 6), it was the concomitant depletion of Ca^2+^ and inhibition of K^+^ efflux which exacerbated hemolysis (Figure 8). This suggests synergistic disruption of volume regulatory mechanisms that could be dependent on basal Ca^2+^ and KCl levels [35].

In conclusion, the current study unveils a previously unrecognized activity of CEP and presents a detailed analysis of the activated cellular pathways which underscores the mechanistic complexity of the erythrocyte response to pharmacological stress. Importantly, protective effects offered by a diverse set of compounds as well as physiological perturbations offer new insights into CEP mode of action which bears significant implications for its proposed role as an anticancer agent. Collectively, these findings position CEP as a promising lead compound for anticancer development while necessitating the imperative to mitigate its hematologic side effects in future preclinical and clinical studies.

## 4. Materials and Methods

### 4.1. Chemicals

All chemicals were purchased from Solarbio Life Sciences (Beijing, China). CEP was prepared as a 20 mM stock solution (12 mg/mL DMSO) which was divided into single-use aliquots and stored at −80 °C. Ringer solutions were prepared as previously described [21].

### 4.2. Experimental Design

The study was approved by the Ethics Committee of King Saud University (E-24-8762), and all volunteers provided written informed consent according to the Declaration of Helsinki. Volunteers were non-smokers and free from chronic disease. RBCs were isolated from 25 fresh blood samples collected in lithium heparin and EDTA vacutainer tubes. Cells were treated for 48 h at 37 °C with and without 5–100 μM of CEP. This range covers the concentrations at which CEP shows anticancer activity as demonstrated in previous studies [4]. Negative and positive control samples were prepared by incubating the cells with DMSO and distilled water, respectively. In inhibitor assays, cells were treated with 100 μM of CEP in the presence and absence of CK1α inhibitor D4476 (20 μM), p38 MAPK inhibitor SB203580 (100 μM), GTPase inhibitor NSC 23766 (100 μM), cyclooxygenase (COX) inhibitor acetylsalicylic acid (ASA; 25 μM), ATP (500 μM), nitric oxide synthase (NOS) inhibitor L-NAME (20 μM), caffeine (500 μM), melatonin (1 μM), ascorbate (1 mM), guanosine (2 mM), adenine (2 mM), PKC inhibitor staurosporin (STSP; 1 μM), MLKL inhibitor necrosulfonamide (NSA; 0.5 μM), urea (25 mM), sucrose (40 mM), and Ca^2+^ chelator BAPTA-AM (10 μM) [36]. The ATP concentration used aligns with intracellular levels [37,38,39,40] and is not toxic based on our own range-finding experiments.

### 4.3. Hemolysis

The absorbance of hemoglobin in the supernatants of control and treated cells was quantified at 405 nm using a LMPR-A14 microplate reader (Labtron Equipment Ltd., Surry, UK) to derive the percentage of cells undergoing hemolysis relative to that induced by distilled water [41]. Lactate dehydrogenase (LDH), aspartate aminotransferase (AST), and AChE activities were measured in the supernatants using the BS-240Pro automated analyzer (Mindray, Shenzhen, China) [42,43].

### 4.4. Eryptosis

Exposure of PS on the cell surface was quantified by 1% annexin-V-FITC (Ex/Em = 488/521 nm) using a Northern Lights flow cytometer (Cytek Biosciences, Fremont, CA, USA) [39] whereas cellular volume was determined by forward scatter (FSC) in 10,000 events. The JSM-7610F ultra-high resolution Schottky field emission scanning electron microscope (JEOL Co., Ltd., Akishima, Tokyo, Japan) was used to illustrate ultrastructural changes [44].

### 4.5. Intracellular Ca^2+^

Cells were labeled with 5 μM of Fluo4/AM for 30 min at 37 °C in the dark, and 10,000 events were analyzed for Ca^2+^ content by flow cytometry [45].

### 4.6. Oxidative Stress

Cells were stained with 10 μM of 2’,7’-dichlorodihydrofluorescein diacetate (H_2_DCFDA) for 30 min at 37 °C in the dark and 10,000 events were analyzed by flow cytometry [46].

### 4.7. Erythrocyte Sedimentation Rate (ESR)

The Westergren method was employed to measure the ESR [47].

### 4.8. Statistical Analysis

Data were analyzed using Prism 9.0 (GraphPad Software Inc., San Diego, CA, USA). All values were expressed as means ± SEM (*n* = 9) derived from three independent experiments. The threshold for statistical significance was *p* < 0.05 for both the *t*-test and one-way ANOVA.

## Figures and Tables

**Table 1:** Summary of CEP toxicity.

Inhibitor	PS Externalization	Cell Shrinkage	Hemolysis
SB203580			
D4476			
Acetylsalicylic acid			
Necrosulfonamide			
Melatonin			
NSC23766			
ATP			
BAPTA-AM			
Staurosporin			
L-NAME			
Ascorbic acid			
Caffeine			
Adenine			
Guanosine			
Polyethylene glycol 8000			
Sucrose			
KCl			
Urea			
Ca^2+^ elimination			
Glucose			

Red indicates stimulation, green indicates inhibition, grey indicates no effect, and uncolored indicates endpoints were not tested.

## Data Availability

The data supporting the findings of this study are available from the corresponding author upon reasonable request and subject to approval by King Saud University.

## Supplementary Materials

The following supporting information can be downloaded at https://www.mdpi.com/article/10.3390/ijms26157250/s1.
