# Supplementary material for: Cepharanthine Promotes Ca2+-Independent Premature Red Blood Cell Death Through Metabolic Insufficiency and p38 MAPK/CK1α/COX/MLKL/PKC/iNOS Signaling
Source: Int J Mol Sci. 2025 Jul 27;26(15):7250. doi: 10.3390/ijms26157250 (PMC12347933; doi:10.3390/ijms26157250)
Supplement: Supplementary file 1 [file ijms-26-07250-s001.zip › ijms-3745374-supplementary.pdf]

Supplementary material for "Cepharanthine Promotes Ca^{2+}-independent Premature Red Blood Cell Death through Metabolic Insufficiency and p38 MAPK/CK1*α*/COX/MLKL/PKC/iNOS Signaling" by Alruwaili *et al*.

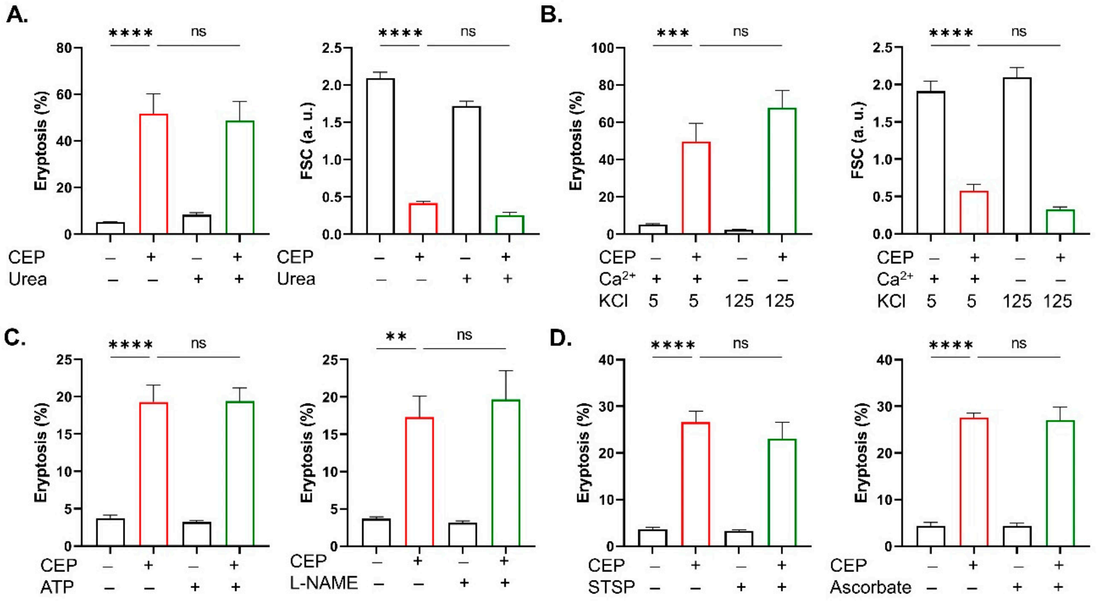

Figure S1: Interventions without impact on CEP-induced eryptosis. PS exposure and FSC with and without (A) urea (25 mM), (B) 5 and 125 mM KCl in absence of Ca^{2+}, (C) ATP (0.5 mM), (D) L-NAME (20 µM), (E) STSP (1 µM), and (F) ascorbate (1 mM). Results are shown as means ± SEM (n = 9) as analyzed by one-way ANOVA. No statistical significance is indicated by ns whereas **(p <0.01), ***(p <0.001), and ****(p <0.0001). CEP concentration: 100 µM. Incubation time: 48 h.

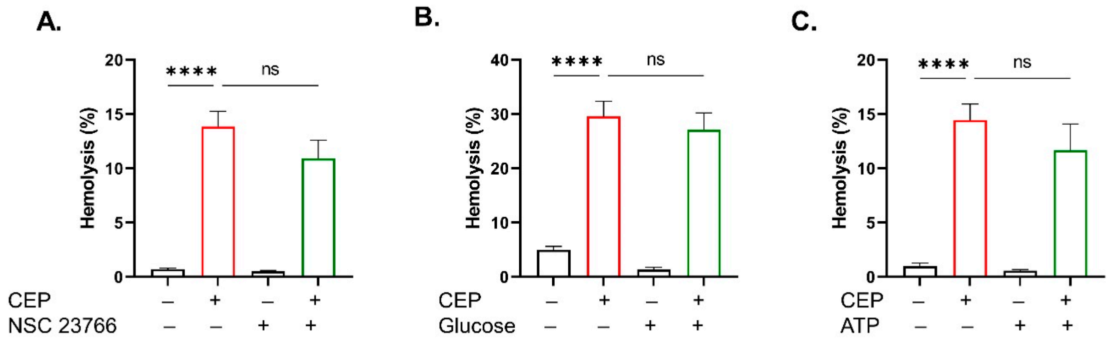

Figure S2: Factors with no effect on CEP-induced hemolysis. Hemolysis with and without (A) NSC 23766 (100 μM), (B) glucose (25 mM), and (C) ATP (0.5 mM). Results are shown as means $\pm$ SD ($n = 9$) as analyzed by one-way ANOVA. No statistical significance is indicated by ns whereas ****(p <0.0001). CEP concentration: 100 μM. Incubation time: 48 h.
